# A simple way to allow continuous ventilation during tracheostomy

**DOI:** 10.1308/003588412X13373405387050g

**Published:** 2012-10

**Authors:** G Chawdhary, P Silva, A Lamyman

**Affiliations:** Oxford University Hospitals NHS Trust,UK

During open tracheostomy, formation of the tracheal window may puncture the endotracheal tube cuff inadvertently. This causes loss of the controlled airway, necessitating rapid and accurate tracheostomy tube placement. This could induce anxiety in both the anaesthetist and surgeon. In our practice we advance the endotracheal tube before fashioning the window so the cuff is distal to the tracheostomy site. Mucosal damage is avoided by deflating the cuff slightly before advancement and subsequent reinflation. There is a risk of unilateral ventilation (albeit only for a short time) if the tube is advanced too far. This technique allows more controlled tracheostomy placement.

**Figure 1:**
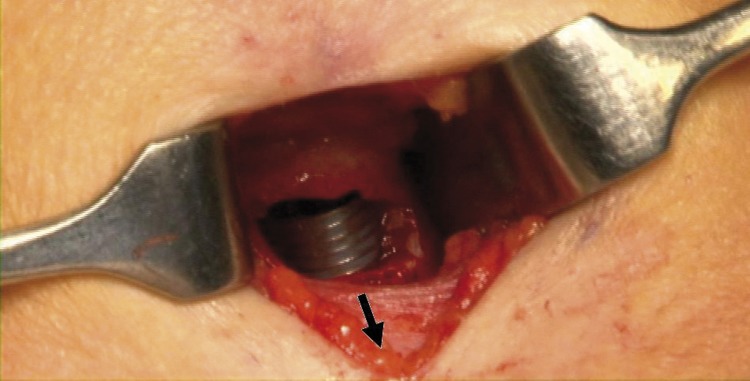
Operative photograph showing tracheostomy: The endotracheal tube has been advanced caudally to ensure the cuff is safe when forming the tracheal window.

